# The Influence of Selected Local Phenomena in CFRP Laminate on Global Characteristics of Bolted Joints

**DOI:** 10.3390/ma12244139

**Published:** 2019-12-10

**Authors:** Krzysztof Puchała, Elżbieta Szymczyk, Jerzy Jachimowicz, Paweł Bogusz, Michał Sałaciński

**Affiliations:** 1 Institute of Mechanics and Computational Engineering, Military University of Technology, gen. Sylwestra Kaliskiego Street 2, 00-908 Warsaw, Poland; elzbieta.szymczyk@wat.edu.pl (E.S.); pawel.bogusz@wat.edu.pl (P.B.); 2 Air Force Institute of Technology, Ksiecia Boleslawa Street 6, 01-494 Warsaw, Poland; michal.salacinski@itwl.pl

**Keywords:** CFRP laminate, mechanically fastened joints, gradient material model

## Abstract

High specific mechanical properties of composites are the reason for their use in various fields, e.g., the aerospace industry. Mechanical joints are still used in the aerospace industry to assembly large aircraft structures. The properties of laminate around the hole can be, however, weakened, compared to their nominal values as a result of a drilling process or cyclic loading. This paper aims at the classification and analysis of imperfections affecting mechanically fastened joints in a laminate structure. A method of modeling the hole vicinity, a gradient material model, as well as the numerical and experimental estimation of laminate deterioration in this area, were proposed and analyzed. Comparative analysis of numerical and experimental results based on displacements of the testing machine grip and the extensometer length confirmed the aforementioned results as consistent in linear ranges. Therefore, joint characteristics obtained based upon measurement of the grip displacement and the ratio of stiffness in linear ranges are sufficient to determine the parameters of a gradient material model. Some imperfections resulting from, e.g., asymmetry, were included in the gradient material model; thus, the obtained weakening of laminate properties in the hole vicinity can be overestimated. Therefore, further analyses of the gradient material model for laminate structures are necessary.

## 1. Introduction

**Figure 1:**
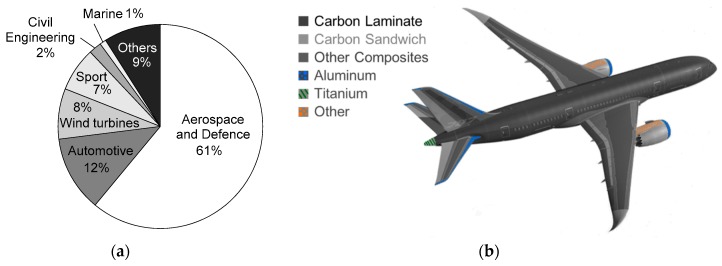
Use of composites: (**a**) Carbon Composite material market (redrawn from [8]); (**b**) Materials used in a Boeing 787 (redrawn from [9])

Advantageous mechanical properties of composites together with their lower mass in comparison to conventional materials [1,2,3] are the reasons for their use in various branches of industry, e.g., aerospace, military, high-tech car, energy turbines and other domains, such as pipeline repairs [4,5] (Figure 1a). Beneficial features of laminates, including properties tailoring, i.e., adapting their configuration to service load, create a possibility to use them for primary structures, e.g., frames, longerons, stringers, ribs and coverings (Figure 1b). Thin-walled aircraft elements, including laminate panels, are connected and reinforced with stiffeners in order to build an airframe. Assembling a whole structure from simpler elements also creates a possibility to repair and replace a selected part if it is necessary.

Mechanically fastened joints (riveted, bolted, etc.) belong to the most reliable methods of connection, and despite some disadvantages, especially in laminate structures, they are still used beside adhesive and hybrid joints [6,7]. However, the holes, necessary for mechanical joints, constitute free edges and lead to stress concentration. Additionally, they are subjected to point loads. These phenomena lead to severe stress concentrations in the area of mechanical joints, and therefore, they demand special attention during the design, manufacturing and service stages.

Many papers deal with mechanical joints, especially in a laminate structure [10,11,12,13,14,15,16,17], and conclude that mechanical joints still undergo both the experimental and numerical studies in order to improve their behavior. Various methods for modification of the hole vicinity are presented and analyzed [18,19,20,21]. Local FML, analyzed in refs. [3,22,23,24], is one of the aforementioned methods. In the paper, the comparison of both the experimental and the numerical results of CFRP laminate in mechanical joints is performed in the aspect of laminate modeling and a possible further use in the modeling of FML.

There are different methods and levels for laminate modeling, due to a complex laminate structure and different aims of analysis [25,26,27]. Despite a continuous increase in the computing power and performance (calculation ability) of numerical algorithms, the modeling of mechanically fastened joints (riveted, bolded, etc.) of CFRP laminate is still a challenging task. On the one hand, this difficulty is caused by a complex laminate structure (different stiffness of components, various modes of laminate failure, as well as notch and point load sensitivity of laminates [1,28,29]). On the other hand, it results from the nonlinearity of the contact problem and size of the connected parts of the structure.

The development of a suitable model of the joint requires identification of parameters affecting its stiffness and load carrying capacity (i.e., strength of the joint) as well as classification of those parameters with respect to an adequate criterion (adequately to the aim of analysis). The paper presents a classification and discussion of the selected parameters.

In order to obtain materials of high quality, characterized by repeatable properties, composite aircraft structures are made of preimpregnates (prepregs) using the autoclave technology [30,31]. The stiffness and strength of large structural components assembled with a set of mechanically fastened parts are dependent on the joint quality and influenced by the drilling technology. The properties of laminate around the hole can be weakened/deteriorated compared to their nominal values. This deterioration can be a result of the drilling technology, and on the other hand, the cyclic load during the service of the structure [32,33,34]. Analysis of the hole machining effects can be found in refs. [35,36,37,38,39,40,41]. A literature review on the drilling of composites and damage connected with it is presented in refs. [42,43,44,45,46]. The reverse topography obtained for the hole surface with computer tomography can be found in ref. [47]. The damage analysis, by means of acoustic emission during drilling, is presented in ref. [48]. Furthermore, since the modern aircrafts consist of different materials (e.g., composites and metal alloys), there is a need of drilling the composite–metal stack [49,50].

Delamination is the most studied damage form connected with hole drilling in laminates. However, it can be concluded based on refs. [51,52,53] that other forms of damage also occur and influence mechanical properties of laminate around the hole.

The fundamental methods for determining the material properties and characteristics of structural components (including joints) are experimental tests. They are especially significant in the case of laminates, due to their sensitivity to the manufacturing technology [54], as well as notch and point load sensitivity [55,56,57,58]. 

As it was mentioned above, development of an adequate model of a mechanically fastened composite structure requires an identification of material parameters. Usually, the properties of a single lamina (in the case of an orthotropic material model—nine stiffness and nine stress components) are experimentally determined. In the next step, the properties of laminate, for the selected stacking sequence, can be established based on a Classical Laminate Theory (CLT). Since the actual and calculated properties of laminate usually differ (actual values are usually several percent lower than theoretical ones), they should be experimentally verified and corrected, if necessary, in order to develop the numerical model [59]. Moreover, for the selected stacking sequence of laminate, it is desirable to determine its bearing properties (stiffness and strength) and/or the tensile characteristics of a simple joint. The latter characteristics can be of greater value due to the coupling effects of bearing, by-pass loads and secondary bending in the case of single-lap joints [60,61].

The aim of a series of papers (including refs. [53,62] and this paper) is analysis of the parameters determining the stiffness and strength of laminate in the area of mechanically fastened joints to develop a gradient material model.

## 2. Object of Analysis

**Figure 2:**
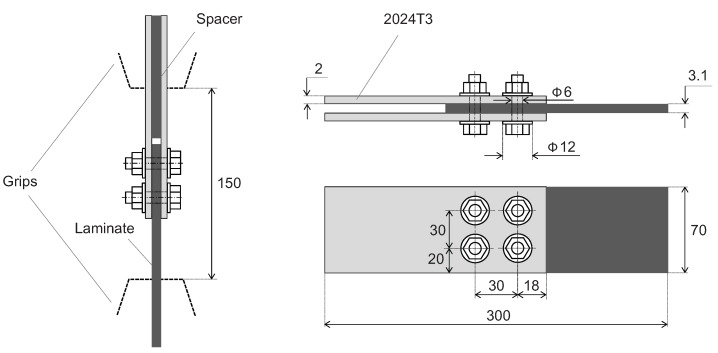
The analyzed double-shear bolted joint.

A double-shear bolted joint with four steel fasteners (Figure 2) is analyzed in the paper. Development of the joint is presented in ref. [63]. The outer sheets are made of 2024T3 aluminum alloy, and the inner element is made of quasi-isotropic CFRP laminate (thermoset epoxy resin reinforced with carbon fibers). CFRP laminate consists of UD layers (HTA/913) and external fabric layers (TR30S twill woven). A stacking sequence of this laminate part is [(0)/0/45/90/45/0/45/90/45/0/90]_s_. The specimen length *L* is 300 mm. The nominal diameter *d* of both the bolt and the hole is 6 mm. Other dimensions of the joint are as follows: The pitch length is equal to 5*d* (30 mm), the specimen width *w* is equal to 70 mm, and the thickness of the aluminum alloy sheet is 2 mm.

Specimens were manufactured by the Air Force Institute of Technology (Warsaw, Poland). The laminate panels were made of preimpregnates in the autoclave technology. The average thickness of a laminate element is 3.1 mm and the standard deviation (SD) is 0.02 mm. Parameters of HTA/913 lamina, TR30S lamina and interfaces are presented in ref. [53]. Pilot holes were made in each part. In the non-cured laminate the initial pilot holes were perforated by the 2.8 mm pins fixed to the mold. Laminate panels (after curing in the autoclave) and aluminum sheets were cut to the specified dimensions with the use of the water-jet. Subsequently, the elements were collected together (as in Figure 2) and pilot holes of 3 mm were drilled. 

Final holes were made in stack of elements in two stages. In the first stage, a 5.8 mm drill was used. In the second stage the holes were reamed with a 6H7 hand reamer. The specimens were assembled with the bolt torque of 10.6 Nm.

The present paper is part of a work intended to improve the performance of mechanically fastened laminates by local reinforcement/hybridization with metal (titanium alloy) sheets. The analyzed joint is a basis to evaluate the reinforcement; therefore, both the basic and reinforced joints should be made with as similar as possible technology. Taking into account the fact that local reinforcement was supposed to be made of titanium alloy sheets, cobalt drills were chosen. A point angle, lip relief angle and a helix angle of the used drills are 140°, 12° and 30°, respectively. The rotational speed was 750 rpm and the feed was 0.1 mm/rev. Dry, compressed air was used as a cooling agent. An average hole diameter measured in the specimens is 6.00 and 6.06 mm, whereas its standard deviation is 0.02 and 0.06 mm for aluminum alloy and laminate, respectively. 

The areas of the initial material failure around the hole were detected with non-destructive testing. An ultrasonic method with C-scan imaging was used with parameters based on refs. [64,65,66]. An average diameter of those areas is 11.25 mm and standard deviation is 1.26 mm [53].

## 3. Numerical Model of Mechanical Joint

Numerical models were developed and analyzed with Mentat^®^ and Marc^®^ code using standard (build in Marc code), finite elements, material models and failure procedures. A nominal (base) numerical model of the joint and its modification leading to satisfactory results (good agreement with the experiment) is presented in refs. [53,62]. The main features of the numerical model are described below. A solid element was used to model all of the components (aluminum sheets, laminate part and fasteners). The results of numerical analysis can be strongly influenced by the mesh density. The proper choice of an element size is especially important in the areas of stress concentration, e.g., holes and point loads. The hole in the joint satisfies both of these conditions. It was noticed that division of the hole circumference into 32 elements is satisfactory for mechanical joints, i.e., further refinement practically does not influence the results [67]. Two times denser mesh (64 elements in the circumferential direction) was used in the analyzed model. The size of elements in the radial direction was chosen so as to achieve proportional discretization in the hole vicinity and a gradual increase along with the radial distance from the hole. In ref. [59], a finite element mesh arrangement and density are also discussed for a single-lap joint, and similar conclusions, concerning the fine mesh around the hole and under the washer, are presented. Additionally, refining of the mesh in the non-overlap region is proposed, due to secondary bending of a single-lap joint. However, the latter effect does not occur in a double-lap joint analyzed in the paper.

Metal components (aluminum alloy sheets and bolts) were modeled as an isotropic elastic –plastic material. Each lamina was modeled as an orthotropic elastic–brittle material using one layer of finite elements. Connections of laminate plies were described using a cohesive material model (zero thickness cohesive elements). Various descriptions of contact were compared in ref. [27], and it was found that a segment to segment analytical contact built in Marc^®^ code gave satisfactory results; therefore, this procedure was taken for the further analyses.

**Figure 3:**
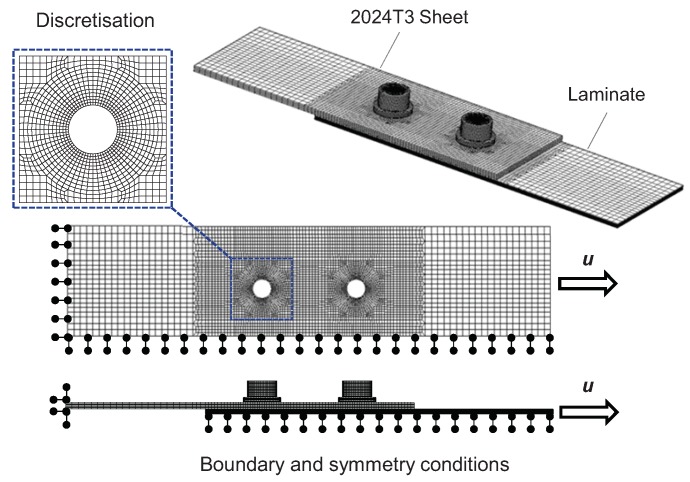
Finite element model—mesh, boundary and symmetry conditions.

The mesh, boundary and symmetry conditions are presented in Figure 3. The left grip edge is fixed and the right grip edge is pulled (displacement u is enforced). Nonlinear, quasistatic analysis was performed using the incremental technique with a constant step size (increment of displacement) equal to 0.01 of the total displacement. It was assumed, due to the joint symmetry, that only a quarter of the joint is necessary to be modeled. Symmetry conditions are frequently used in analyses, e.g., the quarter of the model is presented in refs. [68,69,70]. Taking into account a large number of models and calculations to be carried out for sensitivity analysis and the determining parameters of the gradient material model [53,62], a simplified symmetrical model presented in Figure 3 is also used in the paper. On the other hand, assumption of symmetry is fully justified for a nominal model only, since potential imperfections in the real specimen lead to its asymmetry, which was recognized in the experimental tests. Therefore, an influence of asymmetry on the joint behavior is a subject of further analyses on the development of a gradient material model.

## 4. Analysis of Joint Behavior in the Aspect of Numerical Model Validation

**Figure 4:**
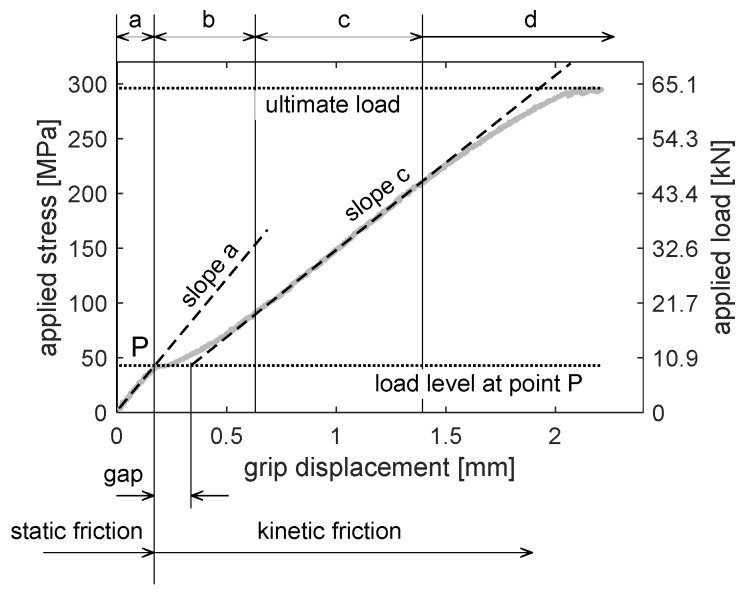
Joint characteristics—load vs. displacement curve: a, proportional range; b, imperfection range; c, main (work) range; d, degradation range.

The typical characteristics of the joint (applied load vs. grip displacement curve) is presented in Figure 4.

The applied stress is calculated as a ratio of the applied load to the cross section of the laminate part. A similar graph is presented, e.g., in ref. [71].

Four ranges *a*-*b*-*c*-*d* can be identified on the graph. In the first proportional/linear range (marked with the letter *a*), the load between parts of the joint is transferred mainly by static friction. In point P, friction becomes kinetic and stiffness rapidly decreases. An imperfection range (marked with the letter *b*) is characterized by a gradual stiffness increase. In this range a joint is straightened, clearance is taken up and other imperfection are levelled (e.g., lack of symmetry, lack of holes concentricity). In the main range (marked with the letter *c*), the curve is almost linear as at the beginning. The load in the connected parts is transferred by both bearing and by-pass stresses. 

In the degradation range (marked with the letter *d*), an evident failure of composite elements occurs, firstly in the bearing area, causing a gradual decrease in the joint stiffness (change of curve slope), and subsequently, in the net cross-section, leading to tearing of the composite element. Simultaneously, the yield stress state can expand in the bearing area of the aluminum alloy sheets. A sequence of the failure of specimen components depends on the joint dimensions and the materials used [63].

Parameters of the joint characteristics, i.e., slope in linear ranges *a* and *c*, size of ranges *b* and *d*, depend upon many factors. In general, due to the assumed permissible tolerances of the manufacturing process, as well as a different kind of imperfections, the results obtained for the nominal model, built using nominal sizes of a joint and nominal material data, do not lead to a satisfactory agreement with the experimental results [53,59,62].

**Figure 5:**
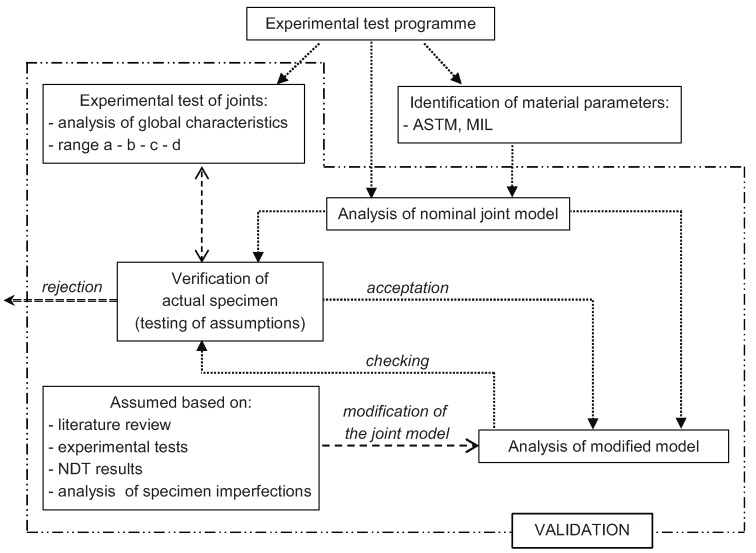
Algorithm of mechanical joint analysis.

In order to study the joint behavior, experimental and numerical analysis was performed. The main stages of analysis are presented in Figure 5.

**Figure 6:**
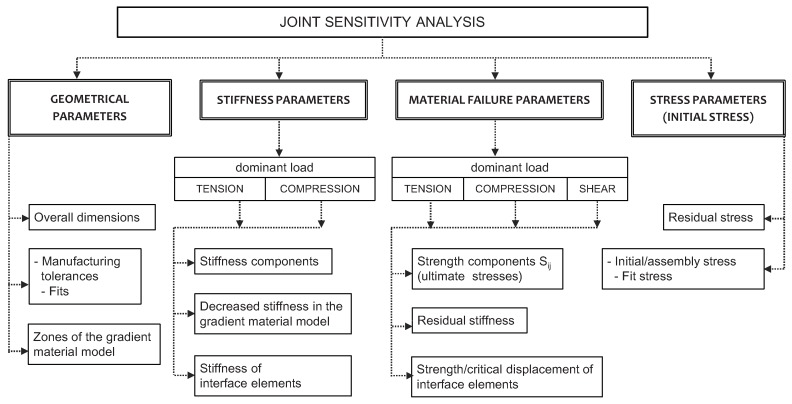
Parameters of joint sensitivity analysis.

After the initial analysis, the following parameters (imperfections), which should be taken into consideration at a validation stage, were indicated (Figure 6):

- geometrical parameters,
- stiffness parameters,
- material failure parameters,
- stress parameters (initial stress).

In the above classification, the shape and material imperfections of a real specimen, including the initial laminate failure caused by drilling, as well as numerical implementation of this failure, i.e., parameters of the gradient material model, are mainly taken into account.

In the group of geometrical parameters, manufacturing tolerances (allowable changes in dimensions of the joint and its components, as well as in the shape and in positional hole tolerances describing fits) are distinguished. Manufacturing tolerances can result in relative displacements of the connected parts, lack of symmetry, non-uniform load distribution, etc. [72].

The joint stiffness depends on the stiffness of elements (components) and their interactions. In ref. [59], different stiffness for the tension and compression of unidirectional CFRP laminate is reported and implemented in the user-defined subroutine in order to improve a numerical model of a single-lap joint. The sensitivity to stiffness components, in the case of a double-lap joint, is analyzed in refs. [53,62]. However, changes in the nominal stiffness by 10% only slightly influence the global response of the joint (global curves); therefore, there should be other reasons for the difference in stiffness between the actual specimen and the nominal model reported in both ref. [53] and ref. [59]. The area of the deteriorated/weakened material around the hole, defined with significantly lower stiffness (compared to the nominal stiffness of the laminate), seems to be a good explanation of the aforementioned difference [53,62]. Both a number and a size of the gradient material model zones are treated as geometrical parameters.

Load carrying capacity of the joint depends on the strength components of CFRP lamina as well as on the yield and ultimate stress of the aluminum alloy. In the numerical model, lamina failure is defined with a failure criterion and a degradation procedure. Failure criteria compare the appropriate components of the stress or strain tensor in the system of material coordinates or their combination with the corresponding values of strength or ultimate strain. When a failure index (a stress or strain ratio of an actual to ultimate value) reaches unity, the degradation of material properties is employed. The residual stiffness defines the ratio of a degraded to a nominal stiffness value. In the paper, the selective gradual degradation procedure (built in Marc^®^ code) is used when the failure, according to the Hashin criterion, occurred. This procedure involves a gradual reduction of the stiffness components to the residual value according to an exponential function of the current failure index [73]. The Hashin criterion and other physically-based criteria (e.g., Puck) allows for the identification of the failure mechanism important for analysis of the complex stress state occurring in a hole vicinity of mechanically fastened joints. However, this paper deals with global, not local, analysis.

In mechanically fastened joints, bearing is coupled with tension in the net section. Thus, forms of joint failure specific for bearing and for tension can be distinguished. In the tension area, stiffness and load carrying capacity after the material failure equals to zero (due to disintegration of material). 

**Figure 7:**
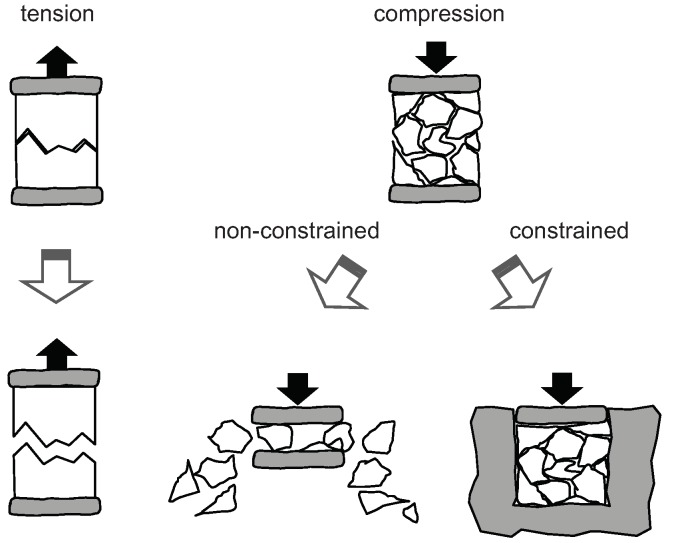
Material behavior in the case of tension and bearing/compression.

Whereas, in the bearing area, the stiffness of the failed/degraded material can be substantially greater than in the tension area, especially if the displacement of material in the transverse direction is constrained (Figure 7).

Interfaces that undergo mainly shear stresses were modeled with the cohesive elements. This created a possibility to perform analysis of the interlaminar shear stress state and simulation of the delamination process. A decrease in cohesive energy (cohesive energy release rate) for the elements placed around the holes was used to describe initial delamination in this area.

In the group of stress parameters, residual and initial stresses were considered. These stresses occur at the manufacturing and assembly stages, including sheet rolling, laminate curing, hole drilling and elements connecting. Manufacturing/assembly stresses can be divided into intentional (desired) and unintentional (undesired). The stresses caused by bolt-torque (and thus washer/nut pressure) belong to the first group. Undesired effects are the result of technological imperfections such as clearances (interferences), lack of joint symmetry, lack of holes concentricity and uneven bearing stress distribution.

**Figure 8:**
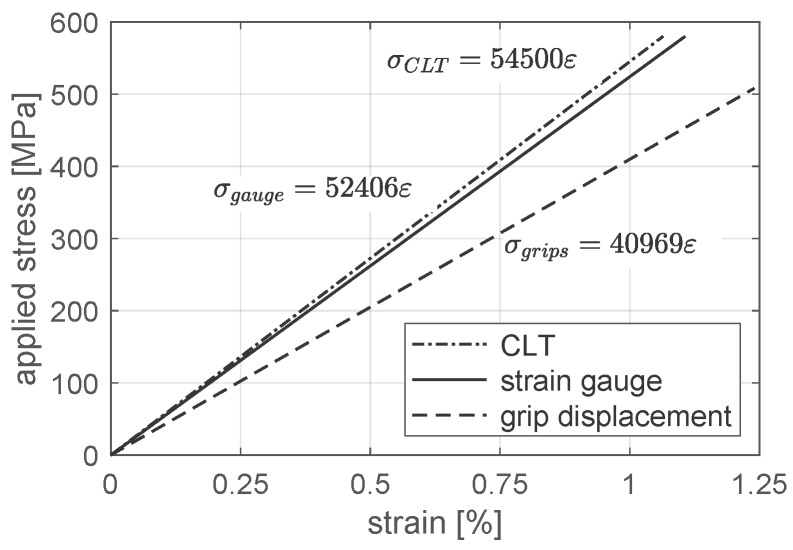
Comparison of stiffness of quasi-isotropic laminate.

The residual stresses remained after the stage of curing the laminate are a result of different thermal expansion coefficients. This phenomenon concerns all laminates consisting of orthotropic plies aligned in different directions; however, it is especially visible for laminates co-curing with metal alloys (typically aluminum or titanium alloys in aircraft structures) [74,75,76]. This effect was not directly involved in the analyzed models; however, it can influence the stiffness and strength of a real laminate specimen. It was observed, during material identification tests, that the longitudinal stiffness of quasi-isotropic laminate is several percent lower than that calculated by means of Classical Laminated Theory (Figure 8).

It is worth mentioning that parameters in the aforementioned four groups are not independent, e.g., stress parameters depend upon geometrical ones (i.e., bolt preload stresses depend on fits); residual stiffness depends on both nominal stiffness and dominant load. Therefore, sensitivity analysis was a time-consuming process, due to couplings among the parameters.

A detailed validation of a numerical model of a bolted joint in the laminate structure, involving clearances, is presented in ref. [77]. Whereas, a literature review shows that parameters of mechanically fastened laminates are still widely studied. The reliability analysis concerning stacking sequence is presented in ref. [78]. An influence of the variation of several parameters on the hybrid bolted-bonded joint behavior is analyzed in ref. [79]. The analyses of the bolt preload as well as of fits including clearances and friction phenomenon can be found in refs. [13,80,81,82,83]. The influence of a hole perpendicularity error on the performance of the composite mechanical joint is presented in ref. [84].

In ref. [59] it is reported that modification of the laminate compression stiffness (from 140 MPa to 130 MPa), mesh refinement in the non-overlap region of the single lap joint, use of the assumed strain formulation with the first order finite elements, as well as modification of boundary conditions by fixing the surface of the clamped region, result in a better agreement between the numerical model and the experimental specimen. Additionally, refinement of the mesh around the hole (under the washer) is applied to improve strain and stress fields. The difference in stiffness decreases from 23.6%, for the base model, to 12.6% for the improved one. The latter difference is explained in ref. [59] mainly by difficulties with a precise measurement of displacement or a nonlinear character of the resin-rich clamped outer surface of the specimen. In the authors’ opinion, this conclusion can be arguable, since various techniques for the measurement of displacement were considered to find the most appropriate solution independent of the clamping effect. Moreover, analysis of the sensitivity of the mechanically fastened CFRP laminate to a number of mechanical and numerical parameters presented in refs. [53,62] shows that deterioration of laminate around the holes can contribute to the characteristics of the joint more than the clamping effect. This conclusion is consistent with the observations presented in refs. [51,52].

Recently, a probabilistic approach to sensitivity analysis has been commonly used. A probabilistic model and sensitivity analysis of several factors of a double-lap, single bolt joint is presented in ref. [85]. Askri et al. [72] proposed a probabilistic analysis of uncertainties (hole-location errors, clearance size and bolt preload) and worst-case analysis of a mechanical four-fastener single-lap joint with the use of a simplified description of the joint and a genetic algorithm. Probabilistic analysis seems to be an appropriate approach to a further development of the weakened material model in the hole vicinity.

Experimental tests are the basis for the identification of material parameters and validation of numerical models. It is worth mentioning that results of experimental tests are dependent on nominal dimensions and material parameters, imperfections of a real specimen, as well as on environmental conditions and measurement techniques.

The object of analysis and nominal parameters are presented in the previous section. Possible imperfections of a real specimen (parameters of joint sensitivity analysis) are listed in Figure 6 and discussed above.

The simplest and most popular method of determining the global characteristics of a tensile loaded joint is to measure the applied force and displacement of the testing machine grip. However, in this case, the total displacement of the grip is a result of specimen compliance as well as compliance (and imperfections) of the fixture (grips compliance, slips and deformation of the specimen in the grip area). An exemplary comparison of the quasi-isotropic laminate stiffness estimated using displacement of the testing machine grip and the strain gauge technique is presented in Figure 8. A similar effect (stiffness 5% lower than nominal) was observed in the laminate part of the tensile loaded joint using a digital image correlation (DIC) technique. This effect is also consistent with the results presented in ref. [59]. 

In ref. [59], several techniques used to estimate the elongation of the free length of the single-lap joint, including determination of a testing machine grip compliance in order to correct stiffness of a numerical model, as well as the use of extensometers, are compared. 

It is reported that the use of linear variable displacement transducers to obtain displacement between steel blocks glued to the side of the specimen at the ends of its free/measurement length was found to be the most appropriate measurement technique.

In ref. [53], a gradient material model is developed based on the global characteristics of the joint, i.e., the applied load versus the displacement of the testing machine grip. In this case, the nominal model does not lead to a satisfactory agreement with the experimental results, mainly due to a grip compliance. Therefore, the numerically obtained characteristics of the joint were scaled/calibrated. The calibration factor was calculated as a stiffness ratio of an experimental to a numerical curve in the proportional range (marked with the letter *a* in Figure 4). Despite the aforementioned calibration, a slope in range *c* was still different for a numerical and an experimental curve (an experimental slope was lower than the numerical one). Therefore, a gradient material model, with an average stiffness significantly lower than the nominal stiffness of the laminate, was defined around the hole. Parameters of the weakened area were estimated with a series of simulations performed to obtain a consistent slope in the range *c*. In other words, in order to exclude the grip compliance and efficiently compare the results of experimental and numerical analysis, two parameters were taken into account in refs. [53,62], i.e., experimentally obtained global characteristics of the joint (based on displacement of the testing machine grip) and a ratio of stiffness in linear ranges *c* and *a* (Figure 4).

The aforementioned scaling/calibration of the joint characteristics does not affect the stiffness ratio *c*/*a*, and should not affect the parameters of the gradient material model as well. However, a shape of the curve, especially in the ranges *b* and *d*, is significantly dependent on calibration, since it is highly nonlinear in these ranges.

Taking into account the aforementioned conditions (deficiency of a curve based on displacement of the testing machine grip), standard and virtual extensometers were used to measure deformation of the joint in the overlap region.

The non-contact optical strain and displacement measurement system Aramis^®^ was involved to obtain a displacement field of selected tensile loaded joints. The measuring area of a specimen was marked with a stochastic black and white pattern. Then, relative displacements of two selected points within this area, used as a virtual extensometer with a 50 mm base, were measured, as shown here in Figure 15c.

The Aramis^®^ system is designed for measurement of deformation on a surface of material during loading. The equipment uses two high resolution CCD cameras of 2358 × 1728 pixels and the digital image correlation (DIC) method to obtain a three-dimensional image sequence.

The aim of the paper is to confirm the correctness of parameters obtained in refs. [53,62] for a gradient material model and in-depth verification of a numerical model of a mechanically fastened joint. Comparative analysis of the numerical and experimental results based on both the displacement of the testing machine grip and length of the extensometer was performed in order to reach this aim.

## 5. Gradient Material Model

**Figure 9:**
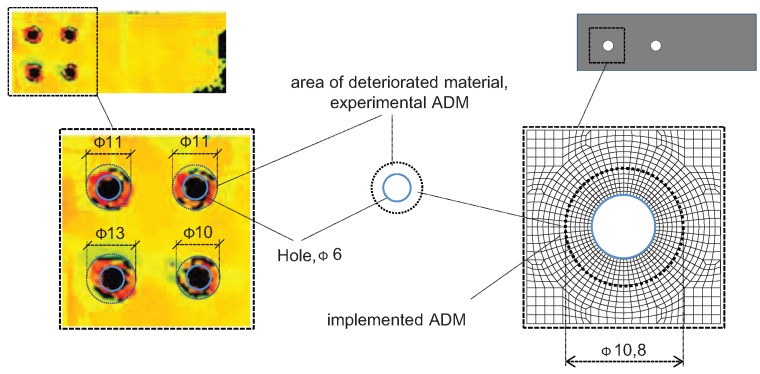
NDT results (C-Scan images) and implemented area of deteriorated material in numerical model [53].

It was identified using the NDT ultrasonic method that the drilling process caused deterioration/changes in the material around the hole. The shape of the area of the deteriorated material (ADM) is irregular; however, it can be approximated using a ring shape with the inner diameter coincident with the hole diameter (Figure 9). Such approximation is more suitable for numerical implementation. The average outer diameter of those areas in the analyzed laminate coupons is equal to about 11 mm. The exemplary results of the NDT tests and corresponding ADM in the finite element model are presented in ref. [53].

**Figure 10:**
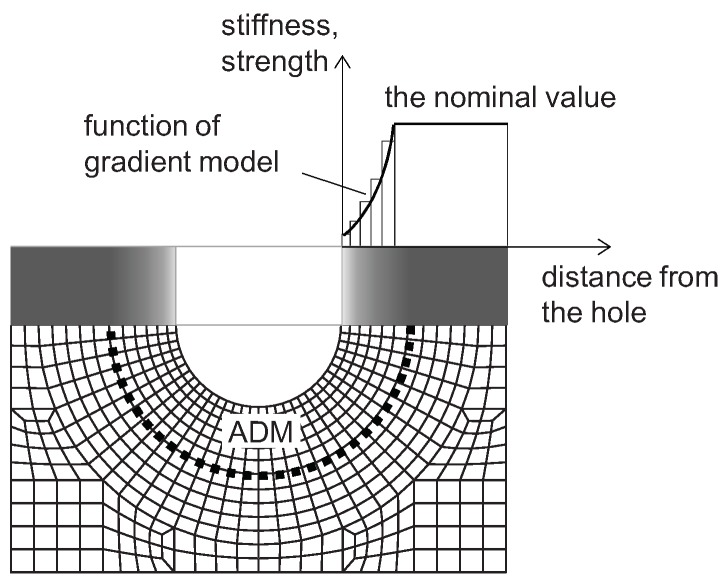
The concept of gradient material model in the hole vicinity.

In refs. [53,62], it was found that the initial delamination only (without any changes of intralaminar properties) did not sufficiently affect the numerical results. This leads to the conclusion that the drilling process caused significant changes of laminae in the hole vicinity, i.e., changes in material properties. In order to describe the laminate deterioration, a gradient material model is proposed in ref. [53]. The function of gradient material properties is unknown and cannot be determined experimentally, therefore, sample functions were proposed to estimate it. The concept of a material gradient model is presented in Figure 10.

**Figure 11:**
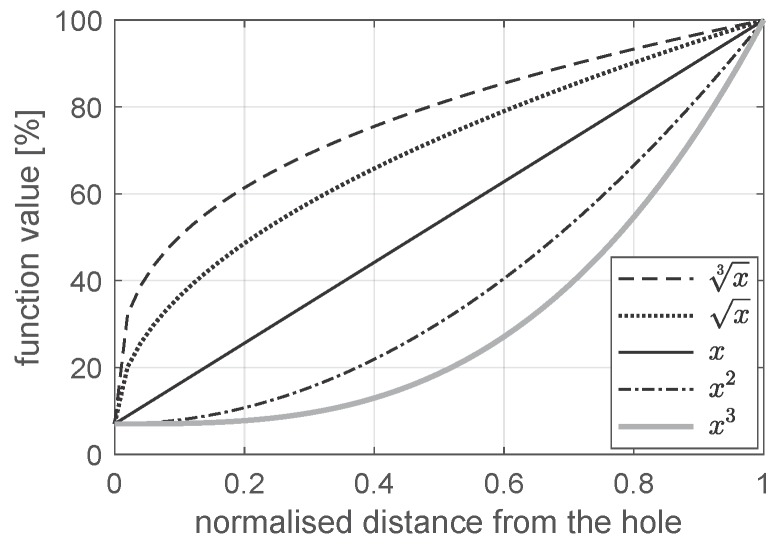
Analyzed sample functions.

Five sample functions were analyzed: cube root, square root, linear, quadratic and cubic function (Figure 11). The sample function describes a stiffness/strength ratio of a current to a nominal value versus the normalized radial distance from the edge of the hole. The normalizing factor is equal to the width of the ADM ring. The minimum value of stiffness/strength at the edge of the hole was established as 7% of its nominal value. This value is both close to zero and high enough to ensure convergence of the numerical calculations. The material parameter at the outer diameter of ADM and beyond this area is equal to its nominal value.

A sample function defines a decrease in the material stiffness/strength compared to its nominal value. An average decrease corresponding to each sample function is shown in Table 1. An average decrease in stiffness/strength (deficiency parameter) can be a measure of the laminate weakness in the hole vicinity.

A series of simulations were performed [53,62] to obtain a satisfactory agreement between the numerical and the experimental results (to estimate stiffness in the hole vicinity).

**Figure 12:**
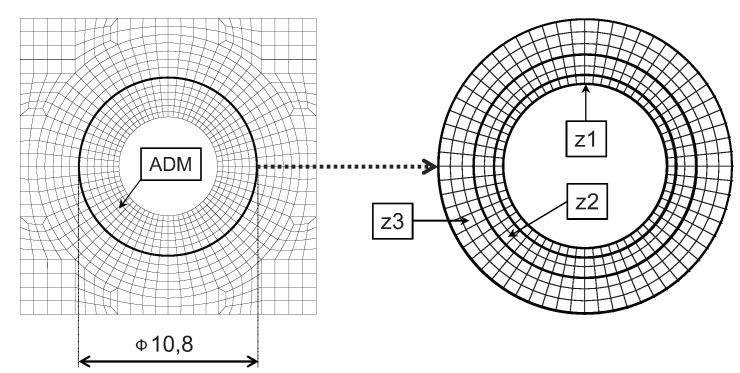
Gradient material model—ADM zones (uneven division).

ADM was implemented in a discrete manner; i.e., the total area was divided into zones with different values of material properties. A value of a sample function corresponding to the middle of a selected zone (of gradient model) was assigned to the whole zone. Several cases of ADM division (in the hole vicinity) were tested [53], and it was found that three zones with a radially increased number of elements (shown in Figure 12) led to the most appropriate results [62], and therefore this case was adopted to further analyses.

**Figure 13:**
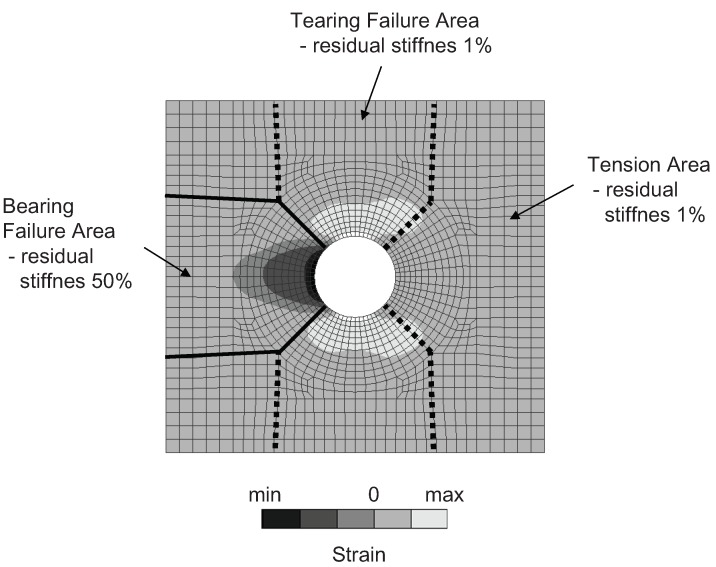
Strain state around the hole. Tension and bearing areas distinguished in the model.

In order to adequately describe the joint failure, two regions, subjected to tension (including tearing) and bearing/compression, were distinguished around the hole (Figure 13).

The residual stiffness for the tension area was assumed as 1% of the nominal stiffness. This value is small enough to be treated as zero. The value of the residual stiffness in the bearing area should be substantially greater, as it is illustrated in Figure 7. This value was found after several trials to gain good agreement with the experimental results in the degradation range *d*.

## 6. Results of Experimental Tests

**Figure 14:**
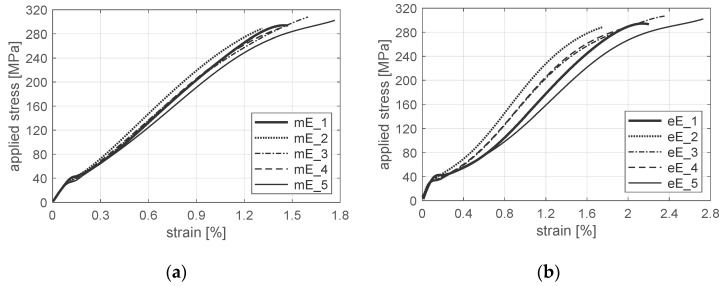
Characteristics of mechanically fastened joints based on: (**a**) displacement of testing machine grip; (**b**) length of extensometer gauge; where prefix m/e—grip displacement/extensometer length, E—experimental results, 1–5—specimen numbers.

Experimental data were collected via a force transducer installed on the transverse beam of the machine, standard extensometers or the digital image correlation (DIC) system Aramis^®^ (serving as virtual extensometer). The results of experimental tests, i.e., characteristics of tensile loaded, mechanically fastened joints, are presented in Figure 14a,b based on displacement of the testing machine grip and length of the extensometer gauge.

**Figure 15:**
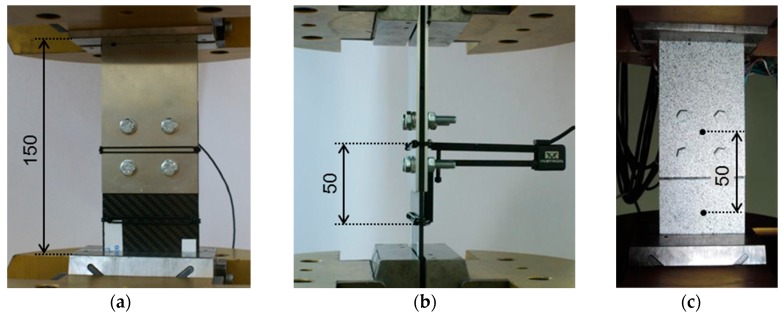
Specimen fixed in the testing machine grips with (**a**), (**b**) extensometer attached across the overlap area and the laminate part; (**c**) virtual extensometer based on Aramis^®^ field

Characteristics of the joint are presented as applied stress vs. strain in order to easily compare the different bases for measuring the displacement. The free length of the specimen fixed in the grips of the testing machine is 150 mm, and the length of the extensometer base is 50 mm (Figure 15).

Comparison of the experimental results, i.e., the load level and the gap (clearance) at point P (Figure 4), as well as stiffness in the ranges *a* and *c*, is presented in Table 2. Specimens numbered as 2, 3 and 4 were initially loaded (preloaded) to 100 MPa, which resulted in smaller imperfections (smaller size of range b) compared to specimens numbered as 1 and 5 (without preload). Those imperfections caused larger deformation of the joint in the overlap region. Strain of the joint obtained, which strain was based on the length of the extensometer, is almost twice as large as the one based on the displacement of the grip (Figure 14a,b). This effect results from both different measurement bases (150 mm and 50 mm) and approximately the same region that is mainly deformed. Stiffness always depends on the measurement base of displacement. It results from a strongly non-uniform strain state in the overlap region.

Analysis presented in the paper is focused mainly on range *c*—the main range of the joint work. In this range, values of average slopes are 23,500 and 19,200 MPa, whereas standard deviations are 1130 and 1948 MPa based on displacement of the grip and length of the extensometer, respectively (Table 2).

## 7. Discussion

As it was mentioned above, displacement of the testing machine grip depends on both joint and fixture compliance, and therefore experimental stiffness was scaled/calibrated (with coefficient 5/3, obtained as a ratio of the numerical to the experimental stiffness in range *a*) in order to compare it to the numerical result. The maximum difference (scatter) of slopes obtained experimentally in range *c* is 5170 (3100 before scaling/calibration) and 5300 MPa, based on displacement of the grip and length of the extensometer, respectively (row difference in Table 2). 

**Figure 16:**
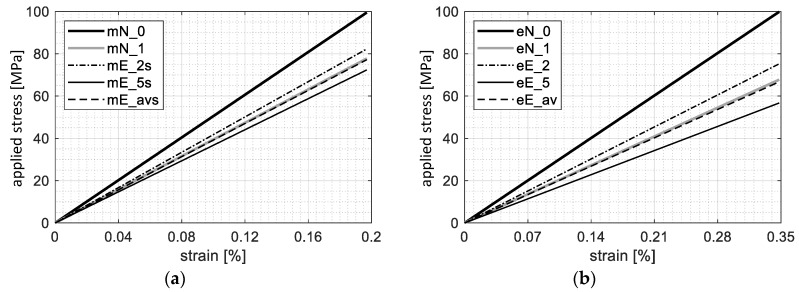
Comparison of the joint stiffness obtained both numerically and experimentally in range *c* based on (**a**) grip displacement, (**b**) extensometer length, where: prefix m/e—grip displacement/extensometer length; N/E—numerical/experimental results; 0—nominal model; 1, 2, 5 —specimen number; av—average stiffness; suffix s—stiffness calibrated with coefficient 5/3

Comparison of joint stiffness obtained both numerically and experimentally in range *c*, based on displacement of the grip and length of the extensometer, is shown in Figure 16. Numerical slope for the nominal model of the joint (N_0) in range *c* is 28,700 MPa. Whereas, a difference of slopes in range *c* between the nominal model (N_0) and the average experimental result (E_av) is about 80% larger than the scatter of the experimental results.

The aforementioned difference between the nominal model of the joint (mN_0, eN_0) and the average (mE_avs, eE_av) or maximum experimental result (mE_2) can be explained by deterioration of the laminate around the hole, which resulted from the drilling process. In ref. [62], based on characteristics of sample No. 1, obtained using displacement of the testing machine grip, the area of the deteriorated material (ADM) around the hole was defined as a gradient material model with a linear function of stiffness and a quadratic function of strength (mN_1). In this case, the result of numerical simulation is in good agreement with both the experimental stiffness of sample No. 1 (as is shown in ref. [62]) and the average experimental value (mE_avs). The results obtained based on displacement of the grip (Figure 16a) are consistent with those based on the extensometer measurement (Figure 16b). Thus, the laminate stiffness around the hole was estimated at 50% of the nominal stiffness, i.e., the average decrease for a linear sample function (compare Table 1). This level is the upper estimation bound since other imperfections can also influence the joint stiffness.

**Figure 17:**
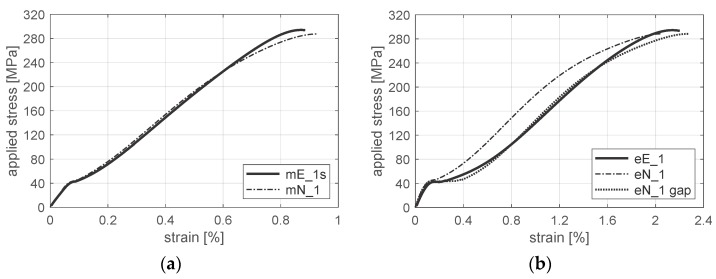
Comparison of the joint characteristics obtained both numerically and experimentally based on: (**a**) displacement of testing machine grip; (**b**) length of extensometer gauge; where prefix m/e—grip displacement/extensometer length; N/E—numerical/experimental results; 1—specimen number; suffix s—grip displacement calibrated with coefficient 3/5; gap—model with increased gap (virtual clearance equal to 0.22% of the extensometer base)

Although the joint stiffness is different for displacement of the grip and length of the extensometer (as is shown in Figure 14 and Table 2), both of them can be used to obtain parameters of a gradient material model and to estimate laminate weakness in the hole vicinity. However, characteristics of the joint based on displacement of the testing machine grip should be scaled/calibrated in order to compare the numerical and the experimental results (Figure 17a). Differences in the joint stiffness depend on various imperfections of a real specimen and the conditions of the experimental test. In refs. [53,62], a relative shift of the hole was used to initially describe all imperfections in range *b* and to obtain good agreement with the experimental results (Figure 17a). Comparison of the numerical and the experimental results, based on the extensometer measurement in the overlap region, revealed that real imperfections were substantially larger than a possible clearance effect (especially for specimens without preload as in the case of specimen No. 1). It was found that in numerical model N_1, virtual clearance (gap) equal to not less than 0.22% of the extensometer base covered all imperfections in the overlap region (Figure 17b).

Summarizing, the aim of the paper is analysis of parameters determining the laminate stiffness and strength in the joint area. The paper presents classification and discussion of the selected parameters affecting the stiffness and load carrying capacity of the joint. The shape and material imperfections of a real specimen, including the initial laminate failure resulting from the drilling, and the initial stresses induced during both the manufacturing and the assemble stage, are classified in Section 4.

Delamination is the most studied damage form connected with drilling a hole in composites/laminates. However, other forms of damage also occur and influence the mechanical properties of laminate around the hole. Therefore, a method for modeling the hole vicinity, namely, a gradient material model, as well as the numerical and experimental estimation of laminate deterioration in this area, was proposed and analyzed.

The laminate stiffness beside the joint area, determined with the DIC technique in the Aramis^®^ system, is 5% lower than the nominal (theoretical) stiffness calculated according to Classical Laminate Theory. A similar result was obtained for the quasi-isotropic laminate during material identification tests, and this effect is consistent with the results presented in literature. Therefore, laminate properties, several percent lower than nominal values, can be taken into account in the numerical model. However, this modification did not cover differences between the nominal model and the real specimen equal to about 50% (Figure 16b).

Weakness of laminate (deterioration of material properties) in the hole vicinity, caused by the drilling process or cyclic loading during the service life of the structure, can be described in the numerical model using a gradient material model. The function of gradient material properties is unknown and cannot be determined experimentally, therefore, the sample functions were used to describe changes in material parameters in the hole vicinity (Figure 11, Table 1). The aforementioned approach is a novelty in the field of the analysis of mechanically connected composite elements.

The results of the experimental tests are dependent on imperfections of the real specimen, test conditions, as well as measurement techniques. Despite nonlinearity of the characteristics of the tensile loaded joint, comparative analysis of the numerical and the experimental results, based on both displacement of the testing machine grip and length of the extensometer, confirmed that the characteristics of the joint, obtained from the measurement of the grip displacement and the ratio of stiffness in the linear ranges *c* and *a*, are sufficient to determine the parameters of the gradient material model and to estimate the weakness of laminate (to find the upper estimation of the laminate weakness) in the hole vicinity.

A simplified symmetrical model used in the paper was sufficient to analyze the global characteristics of the joint; however, it is not able to capture all of the imperfections that resulted from asymmetry of the real joint, particularly in the overlap region (e.g., the clearance shown in range *b* in Figure 17b). Those imperfections can affect to some extent also the joint stiffness in ranges *a* and *c*. If their influence is not negligible, they are included in the gradient material model, and deterioration of laminate properties in the hole vicinity is overestimated.

Analysis of the full model creates a possibility to study the influence of asymmetry on the joint behavior, and is a subject of further works on development of the gradient material model.

## 8. Conclusions

Four ranges *a-b-c-d* were identified on the experimental graphs, i.e., proportional range (marked with the letter *a*), imperfection range (*b*), main work range (*c*) and degradation range (*d*). Numerical slope for the nominal model in range *c* (28,700 MPa) was found about 50% larger than the average experimental result (19,200 MPa). Moreover, the difference of slopes in range *c* (9500 MPa) between the nominal model of the joint and the average experimental result was found about 80% larger than the scatter of the experimental results (5300 MPa). The aforementioned difference can be explained by the laminate weakness/deterioration around the hole resulting from the drilling process.

Weakness of laminate in the hole vicinity was described in the numerical model using a gradient material model with the linear function; i.e., the average laminate stiffness around the hole was estimated at 50% of the nominal stiffness.

Grip displacements are the most easily obtained measurement data, and they are less sensitive to local imperfections than the extensometer data. In the case of the grip displacements, standard deviation of the joint stiffness was about 60% lower than in the case of the extensometer data. Parameters of the gradient material model obtained based on displacement of the testing machine grip and length of the extensometer were almost the same. Therefore, they were successfully verified.

## Figures and Tables

**Table 1:** Comparison of sample functions’ deficiency parameters

No.	Sample Function	Deficiency Parameter (%)
1	cubic root	75.0
2	quadratic root	66.7
3	linear	50.0
4	square	33.3
5	cubic	25.0

**Table 2:** Comparison of the joint stiffness in ranges *a* and *c*.

Specimen No.		Grip Displacement *			Extensometer Length		
	Stress (MPa)	Stiffness (MPa)		Gap (mm)	Stiffness (MPa)		Gap (mm)
	Point P	Range *a*	Range *c*	Point P	Range *a*	Range *c*	Point P
No. 1	42	36100	23900	0.163	39500	18500	0.181
No. 2	31	35700	25100	0.083	42300	21600	0.103
No. 3	30	36500	23200	0.096	39800	19600	0.124
No. 4	31	36900	23300	0.094	35700	19800	0.122
No. 5	31	37400	22000	0.133	38300 **	16300 **	0.170 **
mean		36500	23500	0.114	39100	19200	0.140
std. dev.		665	1130	0.033	2402	1948	0.034
difference		1700	3100	0.080	6600	5300	0.078

* results obtained directly with measurement of the grip displacement (without calibration). ** results obtained using a virtual extensometer based on Aramis displacement field.
